# A unified molecular mechanism for the regulation of acetyl-CoA carboxylase by phosphorylation

**DOI:** 10.1038/celldisc.2016.44

**Published:** 2016-11-29

**Authors:** Jia Wei, Yixiao Zhang, Tai-Yuan Yu, Kianoush Sadre-Bazzaz, Michael J Rudolph, Gabriele A Amodeo, Lorraine S Symington, Thomas Walz, Liang Tong

**Affiliations:** 1 Department of Biological Sciences, Columbia University, New York, NY, USA; 2 Laboratory of Molecular Electron Microscopy, Rockefeller University, New York, NY, USA; 3 Department of Microbiology and Immunology, Columbia University, Medical Center, New York, NY, USA

**Keywords:** fatty acid metabolism, metabolic syndrome, enzyme regulation, enzyme phosphorylation

## Abstract

Acetyl-CoA carboxylases (ACCs) are crucial metabolic enzymes and attractive targets for drug discovery. Eukaryotic acetyl-CoA carboxylases are 250 kDa single-chain, multi-domain enzymes and function as dimers and higher oligomers. Their catalytic activity is tightly regulated by phosphorylation and other means. Here we show that yeast ACC is directly phosphorylated by the protein kinase SNF1 at residue Ser1157, which potently inhibits the enzyme. Crystal structure of three ACC central domains (AC3–AC5) shows that the phosphorylated Ser1157 is recognized by Arg1173, Arg1260, Tyr1113 and Ser1159. The R1173A/R1260A double mutant is insensitive to SNF1, confirming that this binding site is crucial for regulation. Electron microscopic studies reveal dramatic conformational changes in the holoenzyme upon phosphorylation, likely owing to the dissociation of the biotin carboxylase domain dimer. The observations support a unified molecular mechanism for the regulation of ACC by phosphorylation as well as by the natural product soraphen A, a potent inhibitor of eukaryotic ACC. These molecular insights enhance our understanding of acetyl-CoA carboxylase regulation and provide a basis for drug discovery.

## Introduction

**Figure 1:**
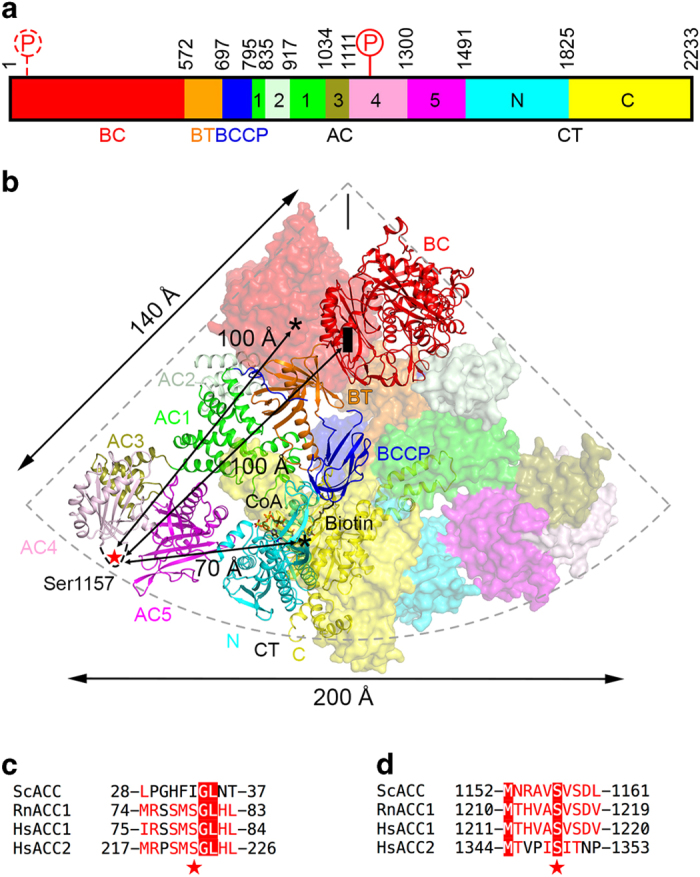
Overall structure of yeast acetyl-CoA carboxylase (ACC) (ScACC). (**a**) Domain organization of ScACC. The domains are labeled and given different colors. The five domains of ACC Central (AC1–AC5) are labeled 1–5. The phosphorylation site in the central region is indicated. The phosphorylation site before the biotin carboxylase (BC) domain core is indicated with the dashed lines, as it is absent in ScACC. (**b**) Structure of the ScACC holoenzyme dimer [7]. One protomer is shown as ribbons, while the other as a surface. The domains in the monomers are colored according to panel (**a**) and labeled. Ser1157 (red star) is located in a loop missing in the structure (dashed lines), and its distances to the BC and carboxyltransferase (CT) active sites (black asterisks) and the BC dimer interface (black rectangle) in the holoenzyme are indicated. (**c**) Sequence conservation near the phosphorylation site before the BC domain core in animal ACCs. Sc: *Saccharomyces cerevisiae*, Rn: *Rattus novegicus*, Hs: *Homo sapiens*. (**d**) Sequence conservation near the phosphorylation site in the central region. The structure figures were produced with PyMOL (www.pymol.org).

Acetyl-CoA carboxylases (ACCs) have crucial roles in fatty acid biosynthesis and oxidation and are promising targets for drug discovery against diabetes, cancer and other diseases [1–6]. ACC carries two distinct catalytic activities, biotin carboxylase (BC) and carboxyltransferase (CT), and its biotin is linked covalently to the biotin carboxyl carrier protein (BCCP). Although bacterial ACCs contain multiple subunits that support these different functions, most eukaryotic ACCs are single-chain, multi-domain enzymes with a molecular weight of ~250 kDa (Figure 1a) and are active as dimers and higher oligomers.

We recently reported the crystal structure of full-length yeast ACC (ScACC) [7] (Figure 1b), revealing the overall architecture of this 500 kDa holoenzyme dimer. The BC and CT domain dimers are located at the top and bottom of the structure, respectively. The unique central region of ACC (Supplementary Figure S1), with five domains (AC1–AC5, AC: ACC Central, Figure 1a), is located at the sides, far away from the two active sites. It likely acts as a scaffold to position the BC and CT dimers correctly for catalysis. BCCP is located in the CT active site. The structures of the BC, CT and BCCP domains alone and those of other biotin-dependent carboxylase holoenzymes have also been reported [1, 8–14].

The catalytic activity of eukaryotic ACCs is tightly regulated by phosphorylation, ligand binding and other means. They are potently inhibited upon phosphorylation by AMP-activated protein kinase (AMPK; known as SNF1 in yeast) [15–17]. The sites of AMPK phosphorylation in human ACC1 include Ser80 (Figure 1c) and Ser1216 (Figure 1d). Residue Ser80 is located prior to the BC domain core, which starts at residue 101. The segment containing phosphorylated Ser80 is recognized by a pocket [18] formed through a large conformational change at the BC dimer interface [7], which makes the BC domain incompatible with dimerization (Supplementary Figure S2). Monomeric BC domain also has a conformational change in the active site region, which would block biotin binding. Therefore, phosphorylation of Ser80 stabilizes a conformation of the holoenzyme in which the BC domain dimer is dissociated, and this monomeric state of the BC domain is catalytically inactive [7]. This new pocket in the dimer interface is also used by soraphen A [19], a polyketide natural product that potently inhibits eukaryotic ACCs [1], suggesting that it may have a similar mechanism of action as Ser80 phosphorylation (Supplementary Figure S2).

In contrast, ScACC does not have a phosphorylation site equivalent to Ser80 in human ACC1 (Figure 1c). Proteomic studies have identified a large number of phosphorylation sites in ScACC [20, 21]. Among these, the segment around Ser1157 appears to be well conserved with the Ser1216 phosphorylation site in human ACC1 (Figure 1d). Ser1157 is located in a loop containing residues 1137–1170 in domain AC4 (Figure 1a, Supplementary Figure S1), and most of the residues in this loop are disordered in the unphosphorylated ScACC holoenzyme structure, although weak electron density was observed for residues 1153–1161 in one of the molecules [7]. This site is ~70 Å from the nearest CT active site and ~100 Å from the nearest BC active site in that structure (Figure 1b).

It has been shown that mutating Ser1157 to alanine leads to increased catalytic activity under conditions where SNF1 is activated [22, 23]. Most recently, the crystal structure of phosphorylated AC1–AC5 of ScACC was reported, with Ser1157 having been phosphorylated during expression in insect cells [24]. The binding mode of phosphorylated Ser1157 was defined and the dynamic behavior of ACC holoenzymes was characterized.

However, currently there are no experimental data showing that SNF1 can directly phosphorylate Ser1157 and whether there are additional sites of SNF1 phosphorylation in ScACC. Here we have developed an *in vitro* phosphorylation system and shown that SNF1 can directly phosphorylate ScACC at Ser1157. We have determined the crystal structure at 2.9 Å resolution of phosphorylated AC3–AC5 domains alone and identified solution conditions that allowed us to directly visualize the ScACC conformation that is observed in the crystal by electron microscopy (EM). EM studies on phosphorylated ScACC reveal dramatic conformational changes, likely owing to dissociation of the BC domain dimer in the holoenzyme. Based on these observations, we propose a unified molecular mechanism for how AMPK phosphorylation (at both sites in animal ACCs) and soraphen A binding regulate the catalytic activity of eukaryotic ACCs.

## Results

### Ser1157 is directly phosphorylated by SNF1

**Figure 2:**
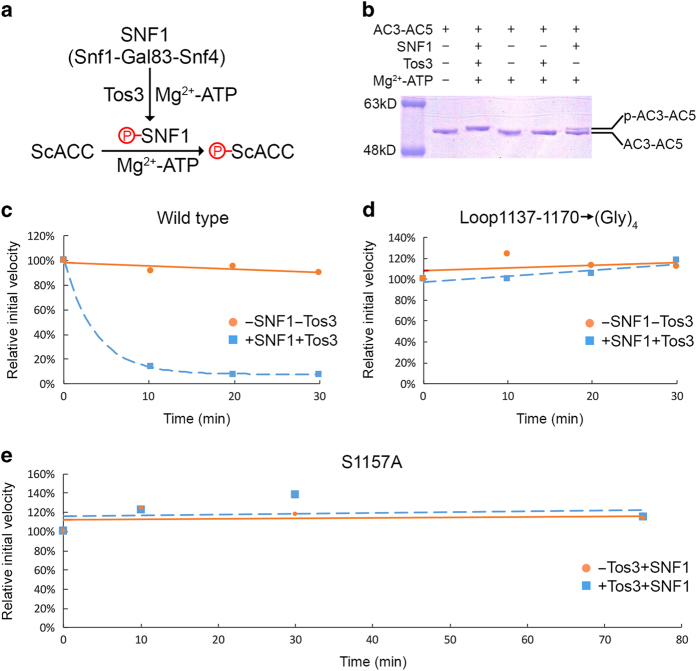
SNF1 directly phosphorylates Ser1157 of yeast acetyl-CoA carboxylase (ScACC). (**a**) Schematic drawing of the *in vitro* phosphorylation system. SNF1 is activated by the upstream protein kinase Tos3, which in turn phosphorylates ScACC. (**b**) Sodium dodecyl sulfate gel shift assay for ScACC phosphorylation, showing a clear shift for the migrating position of domains AC3–AC5 after treatment with SNF1 and Tos3. (**c**) Activity assay for ScACC phosphorylation, showing ~80% loss of the catalytic activity of full-length ScACC after 10 min incubation with SNF1 and Tos3. (**d**) Activity assay showing that the ScACC mutant in which residues 1137–1170 are replaced with a (Gly)_4_ linker is insensitive to activated SNF1. (**e**) Activity assay showing that the S1157A mutant is insensitive to activated SNF1, even after 75 min incubation.

To obtain experimental evidence for the direct phosphorylation of Ser1157 by SNF1, we carried out *in vitro* phosphorylation reactions and monitored their progress by ACC activity assays and/or sodium dodecyl sulfate polyacrylamide gels, noting that a phosphorylated protein generally runs slower than its unphosphorylated counterpart. We overexpressed and purified a SNF1 heterotrimer in *Escherichia coli*, containing the Snf1 catalytic subunit and the Gal83 and Snf4 regulatory subunits, using the same method as that for expressing the SNF1 heterotrimer core (missing primarily the protein kinase domain) [25]. To activate SNF1, we expressed and purified the constitutively active upstream protein kinase Tos3 [26] in *E. coli*. We were then able to produce phosphorylated ScACC by incubation with SNF1 and Tos3 in the presence of ATP and Mg^2+^ (Figure 2a).

We observed a clear shift in the position in sodium dodecyl sulfate gel for domains AC3–AC5 after treatment with Tos3 and SNF1 but not in their absence (Figure 2b), confirming that SNF1 can directly and completely phosphorylate this segment of ScACC (most likely on Ser1157) under the reaction condition tested. Interestingly, SNF1 alone (without Tos3) could also produce a small amount of phosphorylated AC3–AC5, suggesting that it could be weakly active in this buffer. In comparison, Tos3 alone could not produce any phosphorylated AC3–AC5.

For full-length ScACC, we used activity assays to monitor the phosphorylation because a gel shift was difficult to visualize owing to its large size. We observed rapid loss of ScACC activity upon incubation with SNF1 and Tos3, with ~80% of the activity being lost within 10 min (Figure 2c). In comparison, no activity loss was observed, even after 30 min incubation, for an ACC mutant in which residues 1137–1170 were replaced with a (Gly)_4_ linker (Figure 2d). Most importantly, SNF1 had no effect on the activity of the S1157A mutant, even after prolonged incubation (Figure 2e). This result indicates that Ser1157 is the predominant if not the sole SNF1 phosphorylation site in ScACC that is capable of regulating its catalysis.

### Crystal structure of phosphorylated AC3–AC5

**Figure 3:**
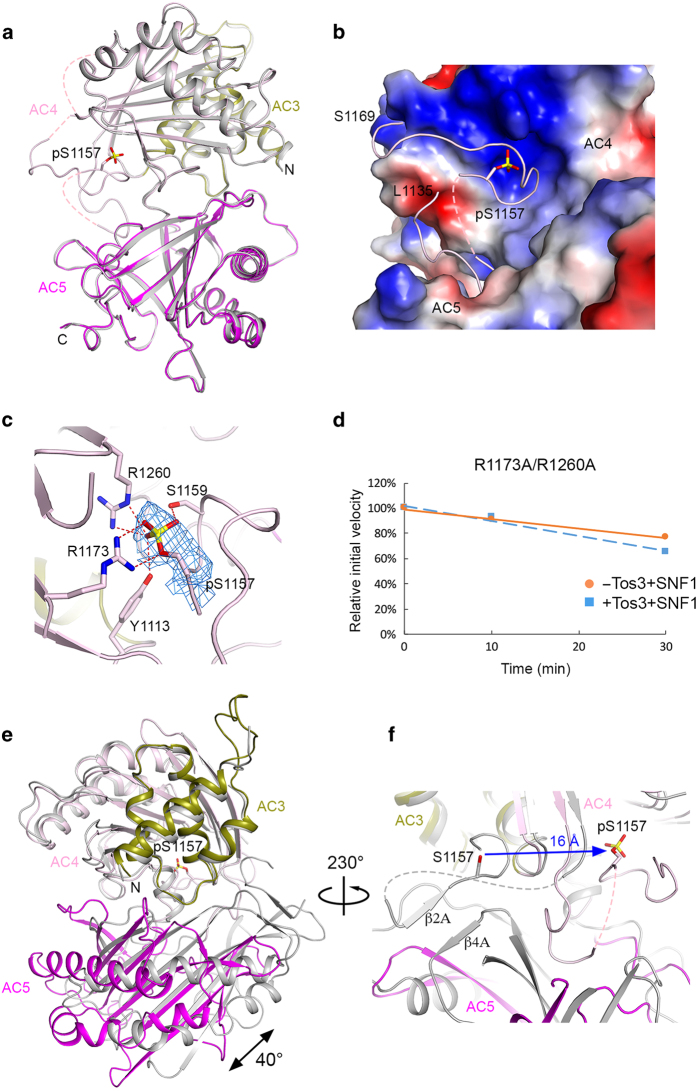
Crystal structure of phosphorylated domains AC3–AC5 of yeast acetyl-CoA carboxylase (ScACC). (**a**) Overlay of the structure of phosphorylated AC3–AC5 (in color) with that of unphosphorylated AC3–AC5 alone (gray). The pSer1157 side chain is shown as stick models. (**b**) Molecular surface of ScACC near the loop containing the pSer1157 residue, colored by electrostatic potential (blue: positive; red: negative). (**c**) The binding site for pSer1157 in domain AC4. Interactions with the phosphate are indicated with dashed lines (red). Omit *F*_o_–*F*_c_ electron density for the phosphate group is shown in light blue, contoured at 3*σ*. (**d**) Activity assay showing that the R1173A/R1260A double mutant is only mildly inhibited by activated SNF1. (**e**) Overlay of the structure of phosphorylated AC3–AC5 (in color) with that of AC3–AC5 in the holoenzyme (gray). Domains AC3–AC4 were used for the overlay, and the large conformational difference for domain AC5 corresponds to a rotation of 40° and is indicated. (**f**) The position of Ser1157 moves by ~16 Å upon phosphorylation. Ser1157 interacts with different residues at the AC4–AC5 interface in the unphosphorylated ScACC holoenzyme structure.

To illuminate the molecular basis for how phosphorylated Ser1157 (pSer1157) is recognized by ScACC, we determined the crystal structure at 2.9 Å resolution of domains AC3–AC5 with Ser1157 fully phosphorylated with our *in vitro* phosphorylation system (Figure 3a, Table 1). The overall structures of the two AC3–AC5 molecules in the asymmetric unit are similar, with root mean square (r.m.s.) distance of 0.45 Å for 402 equivalent Cα atoms between them (Supplementary Figure S3). pSer1157 is observed in both molecules and has essentially the same conformation, but the loop containing this residue is more ordered in one of the two molecules (residues 1137–1143 and 1156–1169 are modeled in one molecule while only residues 1156–1162 are modeled in the other; Supplementary Figure S3) and that molecule will be described further below.

The loop containing pSer1157 is located in a groove at the interface between domains AC4 and AC5, with pSer1157 positioned in an electropositive pocket in domain AC4 (Figure 3b). The phosphate group interacts with the side chains of Arg1173, Arg1260, Tyr1113 and Ser1159 (Figure 3c), and these residues are well conserved among those eukaryotic ACCs that contain this phosphorylation site (Supplementary Figure S1).

To assess the functional importance of this binding site, we created the R1173A/R1260A double mutant and found that its catalytic activity was only mildly inhibited upon treatment with activated SNF1 (Figure 3d). This confirms the structural observations and indicates that this binding site is crucial for the regulation of yeast ACC by SNF1.

The overall structure of the phosphorylated AC3–AC5 is nearly the same as that of unphosphorylated AC3–AC5 alone, with r.m.s. distance of 0.48 Å for their 402 equivalent Cα atoms (Figure 3a). However, there is a large difference in the position of AC5 relative to AC3–AC4 compared with the structure of these domains in the holoenzyme [7]. With domains AC3–AC4 in overlay between the structures of the (phosphorylated) AC3–AC5 and the holoenzyme, the orientation of AC5 differs by a rotation of 40° [7] (Figure 3e). Moreover, unphosphorylated Ser1157 in the holoenzyme structure is located in a different pocket, at the AC4–AC5 interface and ~16 Å away from pSer1157 (Figure 3f), suggesting a large conformational change for the loop containing Ser1157 upon its phosphorylation.

The overall structure of phosphorylated AC3–AC5 is similar to that of phosphorylated AC1–AC5 of ScACC reported recently [24], with r.m.s. distance of 0.63 Å for 391 equivalent Cα atoms between them (Supplementary Figure S4). The binding modes of pSer1157 in the two structures are similar as well (Supplementary Figure S4). However, there are conformational differences between the two structures for the rest of this loop, especially residues 1140–1143, which have well-defined electron density (Supplementary Figure S4). Phe1140 is in contact with Phe1298 in one structure while Phe1143 is in contact with Phe1298 in the other (Supplementary Figure S4).

### Conformational variability for the ACC holoenzyme

To reveal how phosphorylation at Ser1157 inhibits the activity of ACC, we attempted to determine the crystal structure of phosphorylated full-length ScACC but were not able to obtain diffraction-quality crystals after extensive efforts. We then turned to EM.

**Figure 4:**
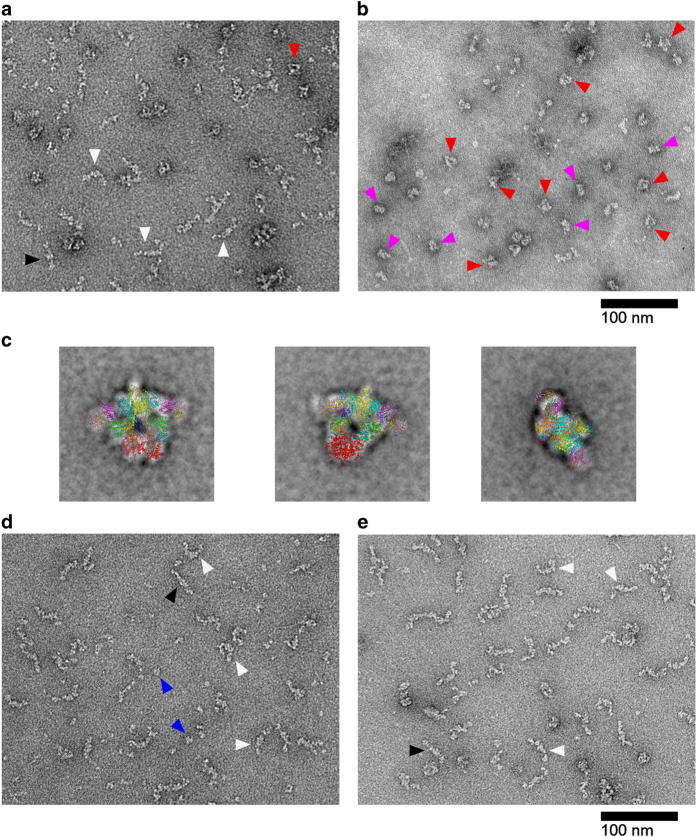
Conformational variability of the yeast acetyl-CoA carboxylase (ScACC) holoenzyme dimer. (**a**) Electron microscopic (EM) image of negatively stained ScACC holoenzyme in the regular protein buffer (20 mM Tris (pH 7.5) and 300 mM NaCl). Predominantly elongated shapes were observed, both straight (black arrowhead) and bent (white arrowhead). A few compact shapes (red arrowhead) are likely similar to the structure observed in the crystal. (**b**) EM image of negatively stained ScACC holoenzyme in a buffer containing 10 mM Tris (pH 7.5), 150 mM NaCl and 100 mM sodium citrate. Mostly, compact shapes were observed, corresponding to front (red arrowhead) and side (magenta arrowhead) views of the structure observed in the crystal. (**c**) Three class averages of negatively stained ScACC in the presence of citrate. The crystal structure of ScACC was overlaid manually to indicate that the EM images are in good agreement with the crystal structure. (**d**) Negative-stain EM image of phosphorylated ScACC holoenzyme in a buffer containing 100 mM citrate. Predominantly elongated shapes were observed. Smaller particles (blue arrowhead) are likely the protein kinases used for phosphorylation (SNF1 and/or Tos3). (**e**) Negative-stain EM image of ScACC holoenzyme with soraphen A in a buffer containing 100 mM citrate. Predominantly elongated shapes were observed.

The crystal structure of the ScACC holoenzyme shows that it adopts the shape of a quarter of a disk (Figure 1b) [7]. To our surprise, when we examined this sample in the regular protein buffer (20 mM Tris (pH 7.5) and 300 mM NaCl) by negative-stain EM, we observed primarily elongated shapes, varying from completely straight to bent, with only a few particles having a compact shape similar to that seen in the crystal (Figure 4a). The elongated shapes are likely caused by the dissociation of the BC domain dimer, and such conformations of the holoenzyme are probably catalytically inactive.

Noting that we were able to observe the compact structure in the crystal, we hypothesized that the crystallization condition may have stabilized that conformation of the ScACC holoenzyme. Consistent with our hypothesis, mostly compact shapes were observed when we prepared negative-stain EM grids with ScACC in the reservoir solution used for crystallization (data not shown). The solution contained 14% (w/v) PEG3350, 4% (v/v) *tert*-butanol and 0.2 M sodium citrate [7]. Further testing showed that citrate alone from this solution was sufficient to produce the compact shape for the holoenzyme (Figure 4b), using a buffer containing 10 mM Tris (pH 7.5), 150 mM NaCl and 100 mM sodium citrate to prepare the EM grids.

We selected particles from the images of the negatively stained specimens collected in the presence of citrate and carried out 2D class averaging. A total of 399 classes were obtained from 13 129 particles (Supplementary Figure S5), many of them bearing resemblance to the crystal structure. In fact, the crystal structure of the holoenzyme could be readily overlaid onto these averages (Figure 4c), giving strong confirmation that we observed the same conformation of the holoenzyme by EM in this buffer condition.

Our EM observations suggest that the ScACC holoenzyme either assumes a defined, compact conformation or a continuum of extended conformations, depending on the buffer conditions. This conformational variability is important for the regulation of this enzyme (see next).

### Phosphorylation of Ser1157 stabilizes inactive ACC conformations

With the establishment of a protocol to visualize the active conformation of ScACC by EM, we next assessed the effect of Ser1157 phosphorylation on the overall structure of the holoenzyme. We observed primarily elongated shapes for phosphorylated ScACC (Figure 4d), even in the presence of citrate, suggesting that phosphorylation of Ser1157 in ScACC inhibits the enzyme by promoting the dissociation of the BC domain dimer.

We proposed earlier that soraphen A inhibits eukaryotic ACC by binding to the BC domain and stabilizing the monomeric, inactive form of this domain in the holoenzyme [7, 19, 27]. We incubated the ScACC holoenzyme with soraphen A and then examined the sample by negative-stain EM in the presence of citrate. We again observed primarily elongated shapes in this sample (Figure 4e), providing direct experimental evidence in support of our model (Supplementary Figure S2).

### S1157A mutation has no obvious effect on yeast cell growth

We next assessed the effect of ACC Ser1157 phosphorylation on yeast cell growth. We obtained haploid strains by sporulation of *Saccharomyces cerevisiae* strain W303D-ACC1^ΔLeu2^ [28] transformed with plasmids carrying either wild-type (WT) *ACC1* or its S1157A mutant. Haploid strains in which one allele of *ACC1* was replaced by a LEU2 cassette could not survive on –LEU plate if no plasmid was complemented, confirming that *ACC1* is essential for survival (data not shown).

**Figure 5:**
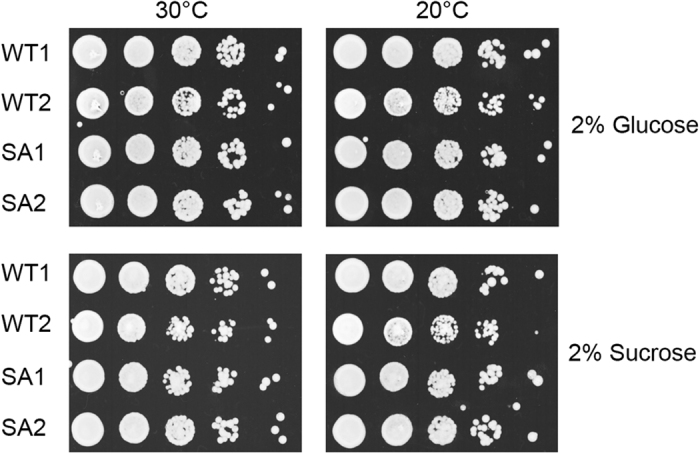
The S1157A mutant has no obvious effect on yeast cell growth under glucose-replete and glucose-limiting conditions. WT1 and WT2: two wild-type strains, SA1 and SA2: two S1157A mutant strains.

The haploid strains complemented with WT *ACC1* or its S1157A mutant were subjected to growth condition analysis. No significant difference was observed between the two strains when they are grown on glucose or sucrose, at either 30 or 20 °C (Figure 5), suggesting that unregulated ScACC activity does not obviously affect cell growth under glucose-limitation conditions. Our observations are consistent with those from earlier studies on this mutant in yeast [22, 23]. Although mutation of Ser1157 did not produce an overall growth phenotype under the condition tested, it did lead to higher ACC activity, elevated fatty acid content and increased biosynthesis of other compounds derived from malonyl-CoA in yeast cells [22, 23].

### A unified mechanism for ACC regulation by phosphorylation

We proposed earlier a model for how phosphorylation of a Ser residue located before the BC domain core (Ser80 and Ser222 in human ACC1 and ACC2, respectively, Figure 1c) and soraphen A inhibit the catalytic activity of eukaryotic ACC [7] (Supplementary Figure S2). Essentially, phosphorylation and soraphen A stabilize a monomeric, catalytically inactive form of the BC domain, and our EM observations on ScACC with soraphen A provide direct evidence that the BC domain dimer has dissociated in the presence of this compound (Figure 4e). Moreover, when the BC domain is isolated away from the rest of the holoenzyme, it preferentially assumes the monomeric, inactive conformation [19].

**Figure 6:**
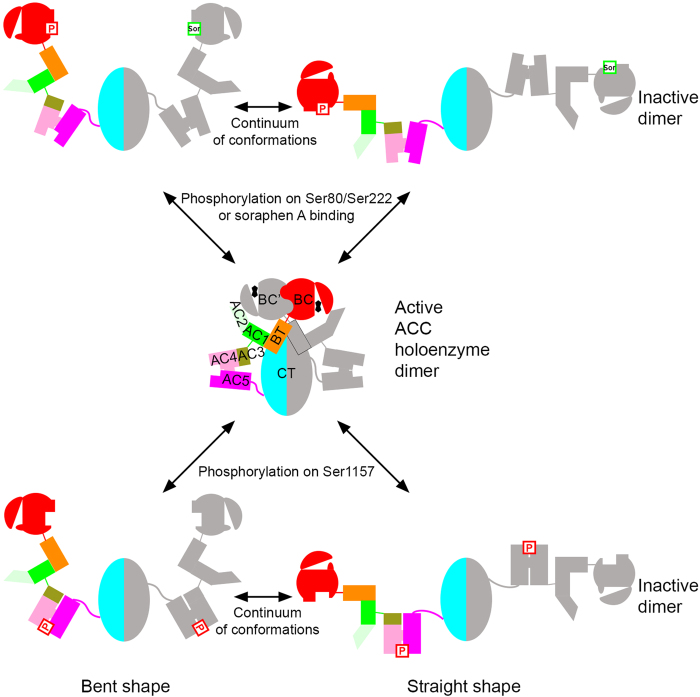
A unified molecular mechanism for the inhibition of eukaryotic acetyl-CoA carboxylases (ACCs) by phosphorylation and soraphen A binding. Phosphorylation at the site before the biotin carboxylase (BC) domain core in animal ACCs (indicated with Ser80 and Ser222 in human ACC1 and ACC2, respectively, labeled P), soraphen A binding (labeled Sor) and phosphorylation in the central region (indicated with Ser1157 in yeast ACC (ScACC)) all stabilize the monomeric form of the BC domain. The monomeric BC domain has large conformational changes in the dimer interface and in the active site region, which blocks biotin binding and thereby catalysis. Biotin is indicated with the fused pentagons in black. Once the BC domain dimer dissociates, the holoenzyme can assume a continuum of elongated conformations, from bent to straight shapes, as observed by electron microscope.

Our EM studies indicate that phosphorylation at Ser1157 in the central region of ScACC also leads to the dissociation of the BC domain dimer. Therefore, we propose a unified molecular mechanism for how phosphorylation at both AMPK sites as well as soraphen A can allosterically inhibit ACC (Figure 6). The central feature of this mechanism is the dissociation of the BC domain dimer into inactive monomers. Once the BC dimer dissociates, the holoenzyme can assume a continuum of extended conformations, explaining the straight and bent shapes observed by EM (Figure 4d and e). This conformational dynamics is also consistent with observations on ACCs in an earlier study [24].

Ser1157 is ~100 Å from the BC domain dimer interface (Figure 1b). How does its phosphorylation lead to the dissociation of this dimer in the holoenzyme? We suggest that the conformational transition between AC3–AC5 domains in the holoenzyme and (phosphorylated) AC3–AC5 alone is the trigger (Figure 3e). The structure of AC3–AC5 alone represents the inactive conformation, which is stabilized by the phosphorylation of Ser1157. This explains why we did not observe any large changes in the structure of AC3–AC5 alone upon phosphorylation (Figure 3a). This situation is reminiscent of that for the BC domain, with the structure of BC domain alone being catalytically inactive and stabilized by phosphorylation on Ser80/Ser222 of human ACCs. On the other hand, phosphorylation of Ser1157 in the context of the holoenzyme would trigger a conformational change for domains AC3–AC5. The 40° rotation of AC3–AC4 relative to AC5 (Figure 3e) is incompatible with the conformation of the holoenzyme observed in the crystal. It would lead to a displacement of the two BC domains in the holoenzyme relative to each other, which would disrupt the dimerization. The exact mechanism how the conformational change for domains AC3–AC5 is propagated to the BC domain dimer will await further studies.

## Discussion

Citrate is a well-characterized activator of animal ACCs and is thought to function through promoting their polymerization into filaments [29]. On the other hand, citrate does not appear to have any effect on the catalytic activity of ScACC (Supplementary Figure S6). Although citrate is a part of the crystallization buffers for full-length ScACC and many of its domains, we have so far failed to find any ordered citrate molecule in our structures. However, our EM studies clearly show that citrate has an impact on the overall structure of ScACC. Citrate may thus have an indirect, non-specific effect by stabilizing the overall structure of the holoenzyme.

We observed the active conformation of ScACC by EM using a buffer solution derived from the crystallization condition. The protocol of using crystallization screening to identify suitable conditions for EM studies could have wider applicability. A sample of interest could be screened (robotically) against the large collection of crystallization buffers currently available, and conditions that give (micro)crystals could then be selected for EM studies.

The ACC CT domain dimer has an extensive interface [30] and represents an anchor in the ACC holoenzyme dimer. On the other hand, the BC domain dimer appears to be a weak link in the ACC holoenzyme dimer. The domain readily distributes between the active, dimeric form (where the holoenzyme would be more compact) and the inactive, monomeric forms (leading to a continuum of elongated conformations of the holoenzyme). Citrate can affect the equilibrium between the two states. It is likely that in cells the compact, active conformation of ACC dominates owing to the various compounds present in the cytosol. The elongated, inactive conformations are observed with our purified, unphosphorylated sample probably because the enzyme was placed in a buffer that is too distinct from the physiological condition.

The structure of CT domain alone is essentially the same as that in the holoenzyme. In contrast, the structure of BC domain or AC3–AC5 domains alone shows extensive differences to that in the holoenzyme. The conformational flexibility is important for the regulation of this enzyme. On the other hand, this also serves as a cautionary tale for the ‘divide-and-conquer’ approach, in that the structures of the isolated domains may not always recapitulate the situation in the context of the full-length protein. At the same time, any conformational differences that are observed may have important roles in the functions of the full-length protein.

Soraphen A inhibits eukaryotic ACCs by taking advantage of their conformational variability. A class of highly potent inhibitors of human ACCs was recently developed based on the soraphen A-binding site [31], indicating a new approach for developing ACC inhibitors. It might be possible that there are other mechanisms of stabilizing the elongated conformations of the holoenzyme. Such compounds would be inhibitors of ACC and could be promising leads for drug discovery as well.

## Materials and Methods

### Protein expression and purification

Residues 22–2 233 and domains AC3–AC5 of ScACC (residues 1 036–1 503) were overexpressed at 25 °C in *E. coli* BL21(DE3) Rosetta cells and purified as described previously [7], with a modification that 2 mM dithiothreitol was included in the gel filtration buffer.

The three subunits of *S. cerevisiae* SNF1 (residues 41–633 of Snf1, 250–418 of Gal83 and 1–322 of Snf4) were overexpressed together, using a polycistronic plasmid built from pET28a (Novagen, Madison, WI, USA) [25], in *E. coli* BL21(DE3) Star cells at 25 °C. Gal83 carried a hexa-His tag. Cells were lysed by sonication in a buffer containing 20 mM Tris (pH 7.5), 150 mM NaCl, 5% (v/v) glycerol, 0.1% (v/v) Triton X-100 and 10 mM β-mercaptoethanol. The complex was purified by Ni-NTA (Qiagen, Hilden, Germany) and gel filtration chromatography (Sephacryl S-300, GE Healthcare, Pittsburgh, PA, USA) in a buffer containing 20 mM Tris (pH 7.5), 150 mM NaCl and 2 mM dithiothreitol.

The segment containing residues 11–460 of *S. cerevisiae* Tos3 was inserted into pET26b (Novagen) and overexpressed in *E. coli* BL21(DE3) Star cells at 25 °C. The recombinant protein with a C-terminal hexa-His tag was purified following the same procotol as that for the SNF1 complex.

### Mutagenesis

Site-specific and deletion mutations were introduced with the QuikChange Kit (Agilent, Santa Clara, CA, USA) and sequenced for confirmation. In deletion mutant Δ1137-1170, residues 1 137–1 170 in domain AC4 were replaced by a (Gly)_4_ linker.

### In vitro phosphorylation, activity and gel shift assays

The *in vitro* phosphorylation reactions contained 4 μM ACC, 0.8 μM SNF1, 0.2 μM Tos3, 2 mM ATP and 5 mM MgCl_2_. The reaction was carried out at room temperature. Incubation at higher temperature (37 °C) or with higher concentration of ATP would induce precipitation.

The catalytic activity of ACC was determined using a coupled enzyme assay, converting the hydrolysis of ATP to the disappearance of NADH [32]. The reaction mixture contained 100 mM HEPES (pH 7.5), 8 mM MgCl_2_, 40 mM KHCO_3_, 200 mM KCl, 0.2 mM NADH, 0.5 mM phosphoenolpyruvate, 0.5 mM ATP, 6 units of lactate dehydrogenase (Sigma, St Louis, MO, USA), 4 units of pyruvate kinase, 100 nM ACC and 0.1 or 1 mM acetyl-CoA. The absorbance at 340 nm was monitored for 60 s.

For gel shift assays, domains AC3–AC5 of yeast ACC (40 μM) were incubated with 1 μM SNF1, 0.2 μM Tos3, 0.75 mM ATP and 0.75 mM MgCl_2_ for 20 min at room temperature before being separated by sodium dodecyl sulfate polyacrylamide gel electrophoresis. Reactions lacking SNF1 and/or Tos3 were included as controls.

### Protein crystallization

Domains AC3–AC5 were incubated in the *in vitro* phosphorylation system with a buffer of 20 mM Tris (pH 7.5), 450 mM NaCl, 2 mM ATP and 5 mM MgCl_2_ at 20 °C for 20 min before crystallization. The final concentration of AC3–AC5 was 2.9 mg ml^−1^ and the molar ratio of AC3–AC5:SNF1:Tos3 was 225:5:1. Crystals of phosphorylated AC3–AC5 were obtained at 20 °C using the sitting-drop vapor diffusion method. The precipitant solution contained 100 mM HEPES (pH 7.5), 3% (v/v) MPD, 2.5 mM sodium citrate and 5% (v/v) glycerol. The crystals appeared within several minutes of setup and were harvested after 2 days. Glycerol was used as the cryo-protectant and crystals were flash frozen in liquid nitrogen for data collection at 100 K.

### Data collection and structure determination

An X-ray diffraction data set of phosphorylated AC3–AC5 domains was collected to 2.9 Å resolution at NE-CAT beamline 24ID-E of Advanced Photon Source, with an ADSC Q315 charge-coupled device detector (Poway, CA, USA). The diffraction images were processed with the HKL program [33]. The crystal belonged to space group *P*2_1_, with cell parameters of *a*=56.4 Å, *b*=93.2 Å, *c*=110.9 Å and *β*=99.6°. There are two molecules in the asymmetric unit, both containing phosphorylated Ser1157. The structure of unphosphorylated AC3–AC5 domains was used as the search model to solve the structure by molecular replacement with the program Phaser [34]. The final atomic model was built with Coot [35] and refined with PHENIX [36].

### EM and image processing

ScACC and phosphorylated ScACC were diluted to 0.05 mg ml^−1^ concentration in buffer A (20 mM Tris (pH 7.5), and 300 mM NaCl) for initial EM studies. Subsequently, samples were incubated overnight in buffer B (10 mM Tris (pH 7.5), 150 mM NaCl and 100 mM sodium citrate) and diluted to 0.05 mg ml^−1^ in 200 mM sodium citrate. To study the effect of soraphen A binding, ScACC was incubated with 100 μM Soraphen A overnight in buffer B and diluted to 0.05 mg ml^−1^ in 200 mM sodium citrate.

Protein samples were prepared for EM by conventional negative staining with 0.7% (w/v) uranyl formate. The negatively stained images were collected at room temperature with a Philips CM10 electron microscope (FEI, Hillsboro, OR, USA) equipped with a tungsten filament and operated at 100 kV. Images were recorded on an AMT XR16L-ActiveVu charge-coupled device camera (Woburn, MA, USA) using a defocus of ~1.5 μm and a nominal magnification of ×50 000.

A total of 13 129 particles were picked manually from 51 charge-coupled device images and windowed into 112×112-pixel images with program e2boxer.py of the EMAN2 software package [37]. After reduction of the particle images to 64×64 pixels, the particles were centered, aligned to each other and classified with the iterative stable alignment and clustering (ISAC) [38] procedure implemented in the SPARX software package [39], specifying 50 images per group and a pixel error threshold of 0.7. After 15 generations of iterative stable alignment and clustering, 399 classes were obtained, accounting for 8 896 particles (67.8% of the entire data set). Averages of these classes were calculated using the original 112×112-pixel images.

### Yeast strains, media and growth conditions

The *S. cerevisiae* diploid strain W303D-ACC1^ΔLeu2^ and pRS426 plasmid carrying the yeast *ACC1* gene were generous gifts from Dr P Gornicki at the University of Chicago [28]. The *ACC1* gene together with its upstream promoter was amplified by PCR from the pRS426 plasmid and inserted into pRS416 vector using the restriction enzymes SacI and XhoI (New England Biolabs, Ipswich, MA, USA).

Rich medium (yeast extract-peptone-dextrose), synthetic complete medium, sporulation medium and genetic methods were as described [40]. The yeast strains used here are listed in Supplementary Table S1. Both WT and S1157A strains were haploid strains obtained by sporulation of W303D-ACC1^ΔLeu2^ transformed with pRS416 plasmids carrying either WT or S1157A mutant *ACC1* gene.

For growth condition assays, serial dilutions of log-phase cultures in synthetic complete-Ura medium were spotted on synthetic complete-Ura or sucrose-Ura (0.67% yeast nitrogen base, 2% sucrose, 1.92 g l^−1^ yeast synthetic drop-out medium supplements without urail, 2% agar) plates for 2 days at 30 °C or for 5 days at 20 °C.

## Figures and Tables

**Table 1:** Data collection and refinement statistics

	*Phosphorylated AC3–AC5*
*Data collection*	
Space group	*P*2_1_
Cell dimensions	
*a*, *b*, *c* (Å)	56.4, 93.2, 110.9
*α*, *β*, *γ*(°)	90, 99.6, 90
Resolution (Å)^a^	50–2.9 (3.00–2.9)
*R*_merge_ (%)	5.1 (50.4)
CC_1/2_	(0.798)
*I*/*σI*	19.2 (2.3)
Completeness (%)	98.3 (99.2)
Redundancy	2.7 (2.7)
	
*Refinement*	
Resolution (Å)	50–2.9
No. of reflections	25 106
*R*_work/_*R*_free_	22.3/28.7
No. of atoms	
Protein	6 626
Ligand/ion	2
Water	0
*B*-factors	
Protein	87.0
Ligand/ion	73.9
Water	—
R.m.s. deviations	
Bond lengths (Å)	0.010
Bond angles (°)	1.3

a One crystal was used for data collection. Highest resolution shell is shown in parenthesis.
