# A unified molecular mechanism for the regulation of acetyl-CoA carboxylase by phosphorylation

**DOI:** 10.1038/celldisc.2016.55

**Published:** 2017-01-17

**Authors:** Jia Wei, Yixiao Zhang, Tai-Yuan Yu, Kianoush Sadre-Bazzaz, Michael J Rudolph, Gabriele A Amodeo, Lorraine S Symington, Thomas Walz, Liang Tong

**Affiliations:** 1 Department of Biological Sciences, Columbia University, New York, NY, USA; 2 Laboratory of Molecular Electron Microscopy, Rockefeller University, New York, NY, USA; 3 Department of Microbiology and Immunology, Columbia University, Medical Center, New York, NY, USA

**Correction to:** *Cell Discovery* (2016) **2**, 16044; doi:10.1038/celldisc.2016.44; published online 29 November 2016

In the initial published version of this article, we omitted the reference to an earlier paper [1] that showed that phosphorylation on Ser1157 was reduced by 50% in *snf1* yeast cells and that the S1157A mutant cells could not grow on media-lacking inositol. The addition of this reference does not affect the results, figures, and conclusion of the paper.
