# Supplementary material for: A unified molecular mechanism for the regulation of acetyl-CoA carboxylase by phosphorylation
Source: Cell Discov. 2016 Nov 29;2:16044–. doi: 10.1038/celldisc.2016.44 (PMC5126230; doi:10.1038/celldisc.2016.44)
Supplement: Supplementary Information [file celldisc201644-s1.pdf]

## Supplementary Table 1

## Yeast strains

| Strain | Genotype | Gene expressed by plasmid | Source |
|---|---|---|---|
| W303D-ACC1$^{\Delta}_{Leu2}$ | *MAT**a**/MATα, leu2-3,112/leu2-3,112 his3-11,15/his3-11,15 ade2-1/ade2-1 ura3-1/ura3-1 trp1-1/trp1-1 can1-100/can1-100 ACC1/acc1::LEU2* | NA* | Ref. 1 |
| WT1 | *MAT**a**, leu2-3,112 his3-11,15 ade2-1 ura3-1 trp1-1 can1-100 acc1::LEU2* | *ACC1*$^{WT}$ | This study |
| WT2 | *MATα, leu2-3,112 his3-11,15 ade2-1 ura3-1 trp1-1 can1-100 acc1::LEU2* | *ACC1*$^{WT}$ | This study |
| SA1 | *MAT**a**, leu2-3,112 his3-11,15 ade2-1 ura3-1 trp1-1 can1-100 acc1::LEU2* | *ACC1*$^{S1157A}$ | This study |
| SA2 | *MATα, leu2-3,112 his3-11,15 ade2-1 ura3-1 trp1-1 can1-100 acc1::LEU2* | *ACC1*$^{S1157A}$ | This study |

* NA, strain was not transformed with any plasmid.

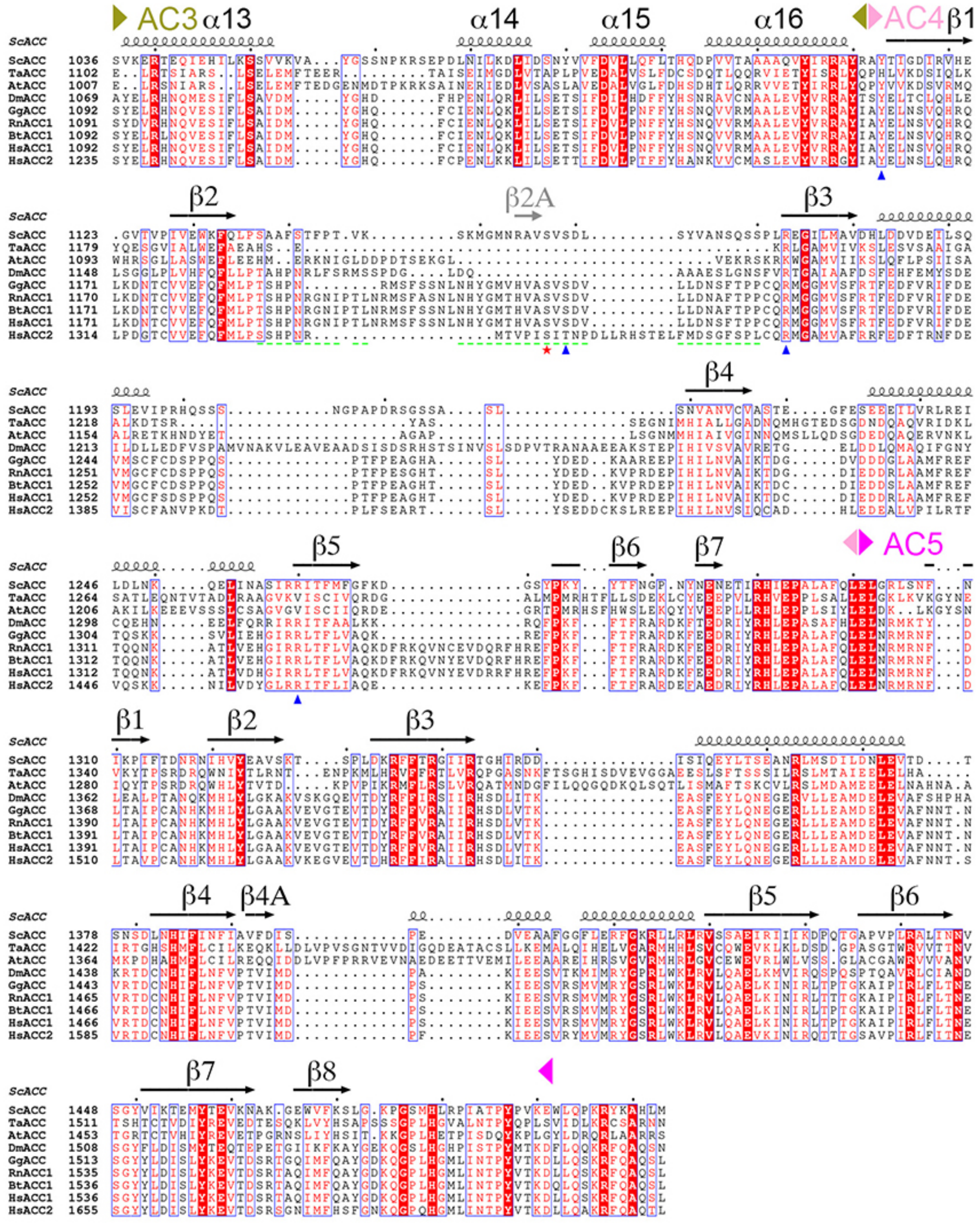


**Supplementary Fig. 1.** Sequence alignment of domains AC3-AC5 of eukaryotic, single-chain ACCs. The secondary structure elements in the ScACC holoenzyme structure are shown and labeled. The site of phosphorylation is indicated with the red star, and blue arrowheads indicate residues in contact with the phosphate. The loop containing the Ser1157 is indicated with the green dashed line. Sc: *Saccharomyces cerevisiae*, Ta: *Triticum aestivum* (wheat), At: *Arabidopsis thaliana*, Dm: *Drosophila melanogaster*, Gg: *Gallus gallus* (chicken), Rn: *Rattus novegicus*, Bt: *Bos taurus*, Hs: *Homo sapiens*. Modified from an output from ESPript [2].

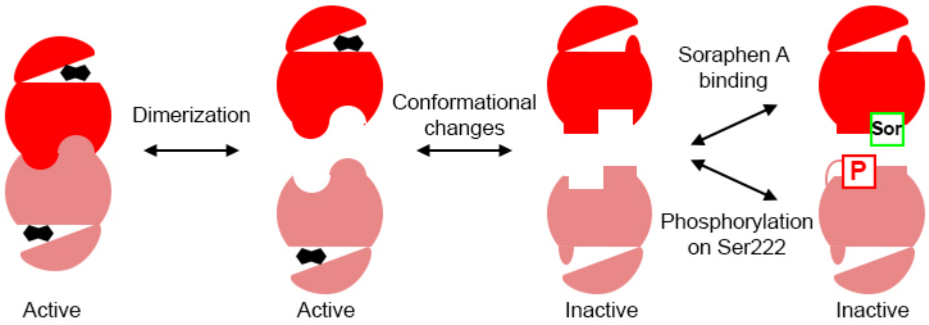


**Supplementary Fig. 2.** A model for the inhibition of ACC by phosphorylation at a site before the BC domain core and by soraphen A binding. Modified from Ref. 3.

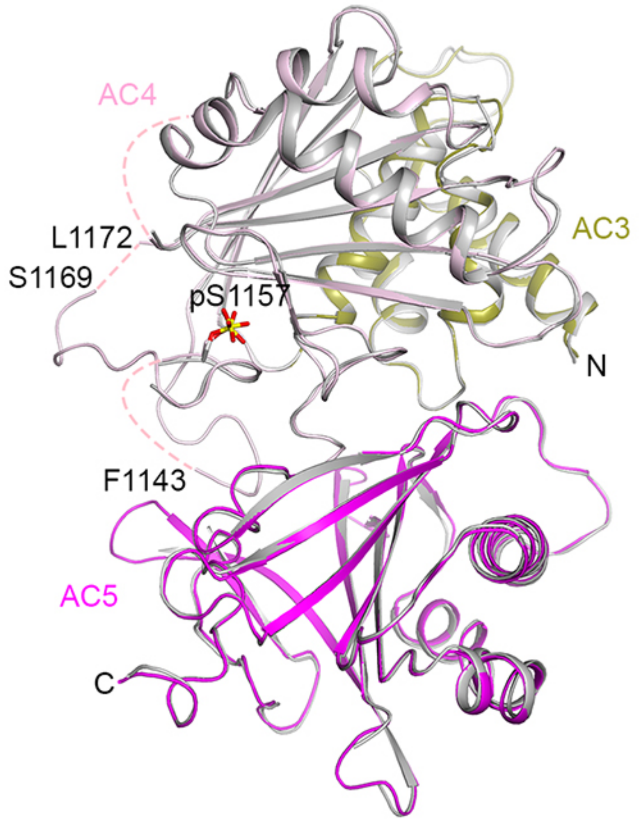


**Supplementary Fig. 3.** Overlay of the structures of the two copies of AC3-AC5 domains in the asymmetric unit. The copy described in the text is shown in color, and the other in gray.

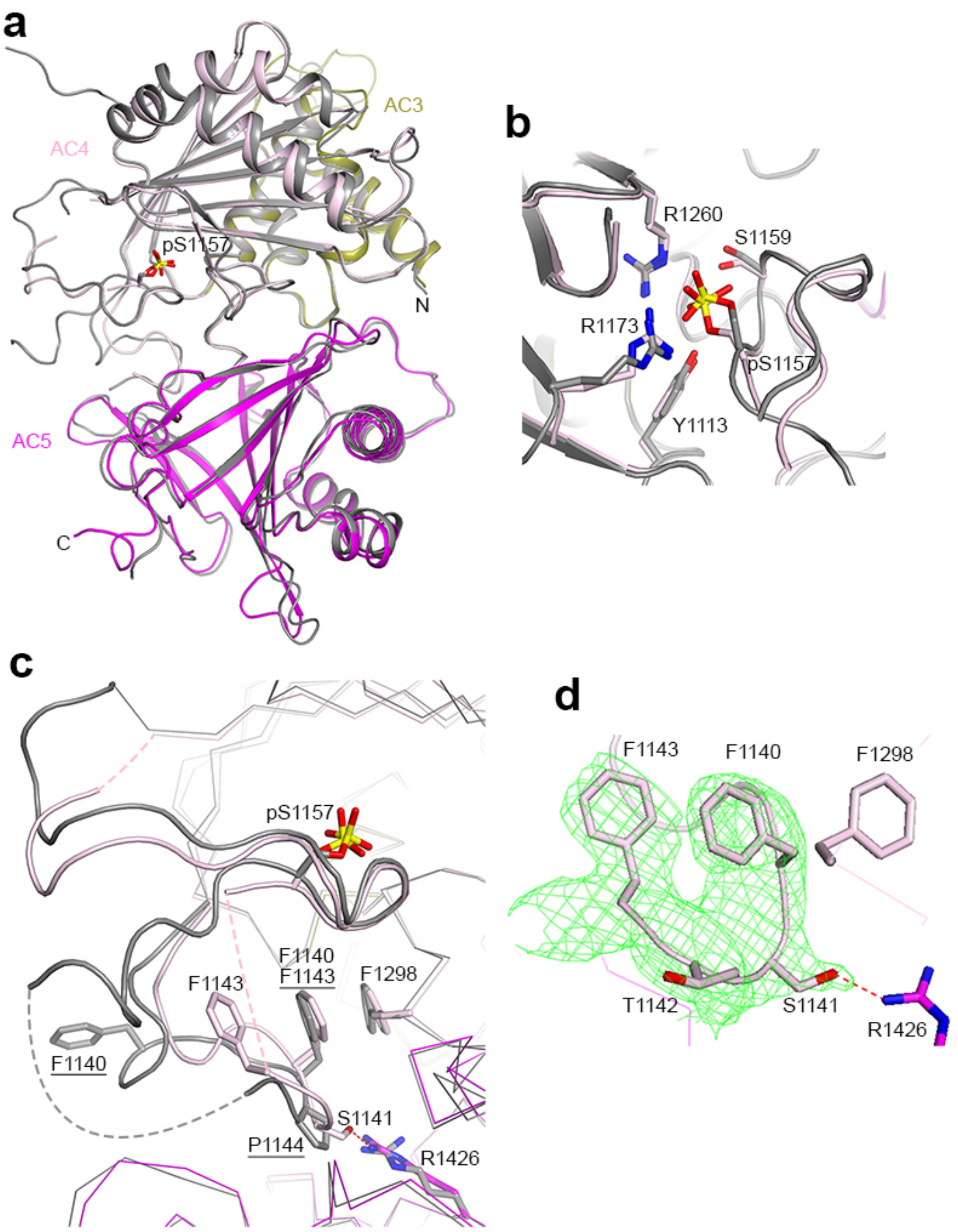


**Supplementary Fig. 4.** Overlay of the structure of phosphorylated AC3-AC5 with that of phosphorylated AC1-AC5. **(a)**. The overall structure of phosphorylated domains AC3-AC5 (in color) is similar to that in phosphorylated domains AC1-AC5 (gray). [4] **(b)**. The pSer1157 binding site has similar conformation in the two structures. **(c)**. A conformational difference for residues 1141-1144 between the two structures. **(d).** Omit $F_o$–$F_c$ electron density for residues F1140-F1143 at 3 Å resolution, contoured at 2σ.

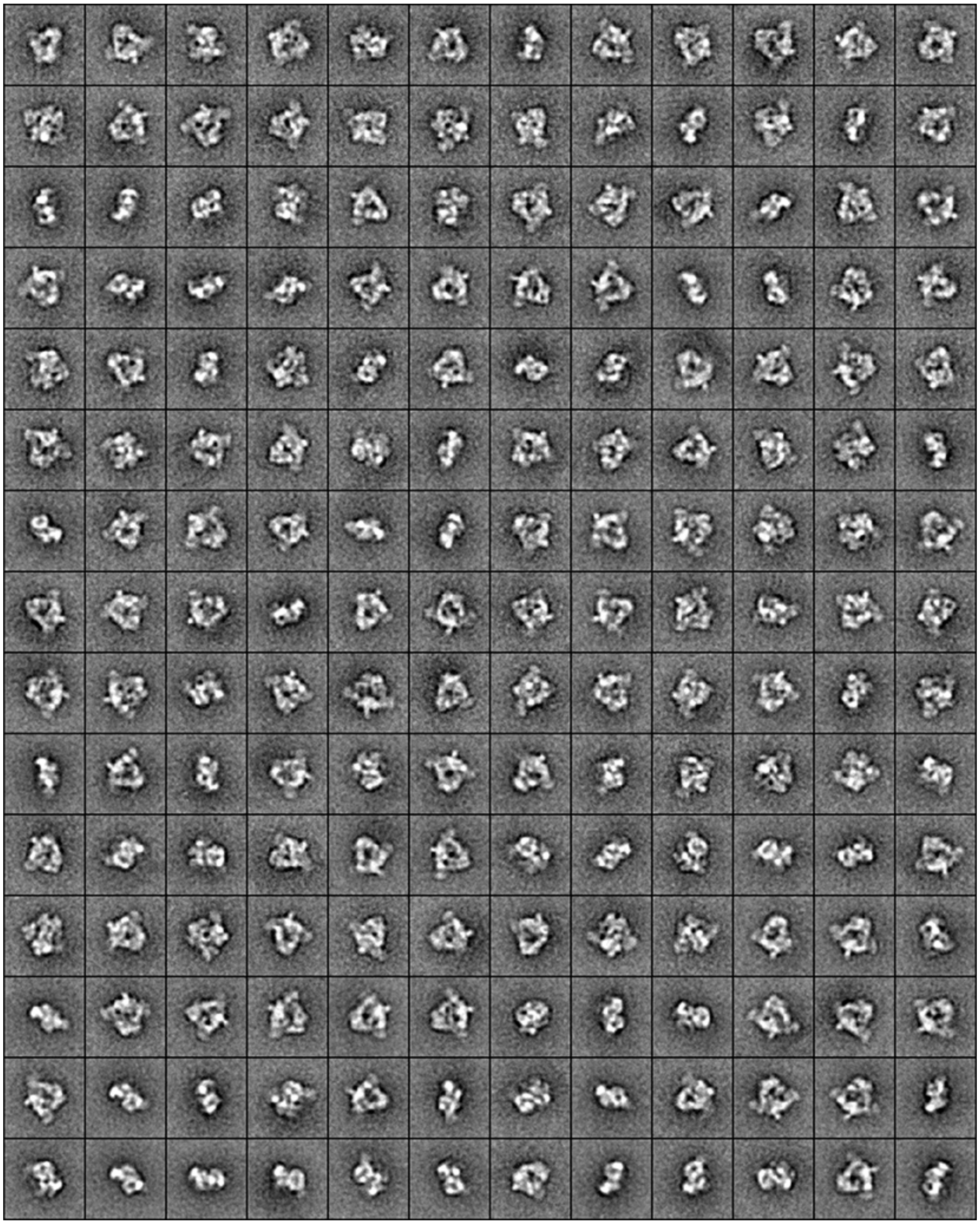

**Supplementary Fig. 5.** 180 representative class averages of negatively stained ScACC. A total of 399 classes were obtained from 15 generations of the iterative stable alignment and clustering (ISAC) procedure [5] implemented in SPARX [6]. These class averages represent 67.8% (8,896 particles) of the entire data set (13,129 particles).

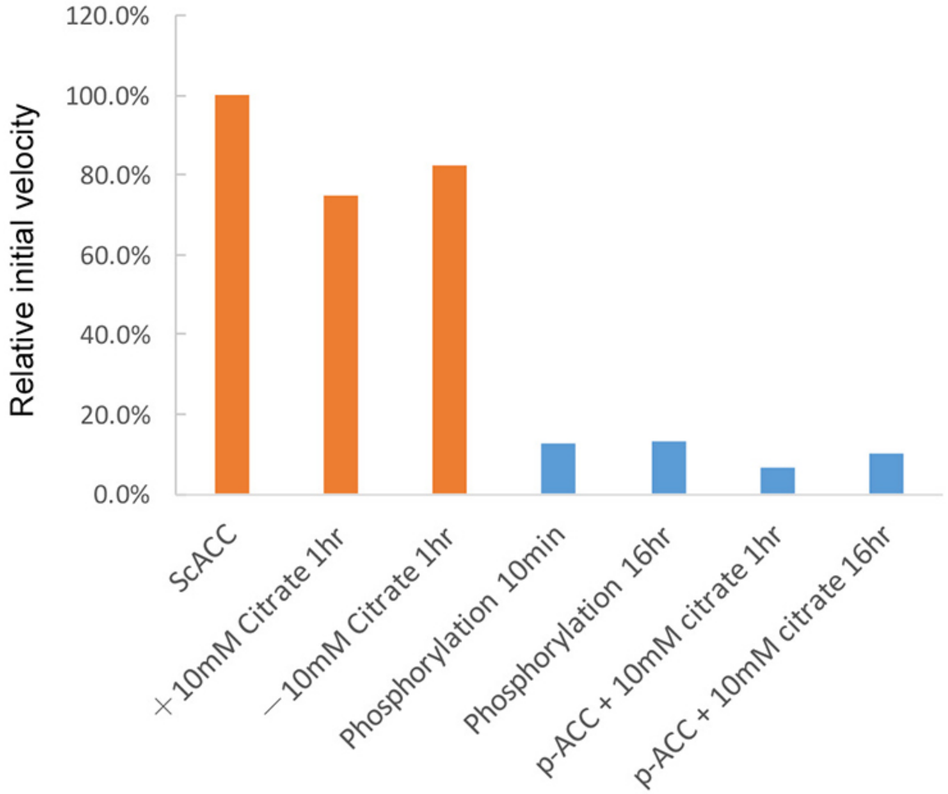


**Supplementary Fig. 6.** Citrate has no apparent effect on ScACC catalytic activity. Citrate does not stimulate the activity of unphosphorylated or phosphorylated ScACC.

## Supplementary References


1. Joachimiak, M., Tevzadze, G., Podkowinski, J., Haselkorn, R. & Gornicki, P. Wheat cytosolic acetyl-CoA carboxylase complements an ACC1 null mutation in yeast. *Proc. Natl. Acad. Sci. USA* **94**, 9990-9995 (1997).
2. Gouet, P., Courcelle, E., Stuart, D. I. & Metoz, F. ESPript: analysis of multiple sequence alignments in PostScript. *Bioinformatics* **15**, 305-308 (1999).
3. Wei, J. & Tong, L. Crystal structure of the 500-kDa yeast acetyl-CoA carboxylase holoenzyme dimer. *Nature* **526**, 723-727 (2015).
4. Hunkeler, M., Stuttfeld, E., Hagmann, A., Imseng, S. & Maier, T. The dynamic organization of fungal acetyl-CoA carboxylase. *Nat. Commun.* **7**, 11196 (2016).
5. Yang, Z., Fang, J., Chittuluru, J., Asturias, F. J. & Penczek, P. A. Iterative stable alignment and clustering of 2D transmission electron microscope images. *Structure* **20**, 237-247 (2012).
6. Hohn, M. *et al.* SPARX, a new environment for Cryo-EM image processing. *J. Struct. Biol.* **157**, 47-55 (2007).
